# Antibody and memory B cell responses to the dengue virus NS1 antigen in individuals with varying severity of past infection

**DOI:** 10.1111/imm.13651

**Published:** 2023-04-19

**Authors:** Shyrar Tanussiya Ramu, Madushika Dissanayake, Chandima Jeewandara, Farha Bary, Michael Harvie, Laksiri Gomes, Ayesha Wijesinghe, Dinuka Ariyaratne, Graham S. Ogg, Gathsaurie Neelika Malavige

**Affiliations:** ^1^ Allergy Immunology and Cell Biology Unit, Department of Immunology and Molecular Medicine, Faculty of Medical Sciences University of Sri Jayewardenepura Colombo Sri Lanka; ^2^ MRC Human Immunology Unit, MRC Weatherall Institute of Molecular Medicine University of Oxford Oxford UK

**Keywords:** antibodies, B‐cell ELISpots, dengue, dengue fever, disease severity, neutralizing antibodies, NS1 antigen

## Abstract

To further understand the role of NS1‐specific antibodies (Abs) in disease pathogenesis, we compared neutralizing antibody levels (Nabs), NS1‐Ab levels, IgG antibody subclass profiles and NS1‐specific memory B‐cell responses (Bmems) in individuals, with varying severity of past dengue. Nabs (Neut50 titres) were assessed using the Foci Reduction Neutralization Test (FRNT) and in‐house ELISAs were used to assess NS1‐Abs and NS1‐Ab subclasses for all four DENV serotypes in individuals with past DF (*n* = 22), those with past DHF (*n* = 14) and seronegative (SN) individuals (*n* = 7). B‐cell ELISpot assays were used to assess NS1‐specific Bmem responses. 15/22 (68.18%) individuals with past DF and 9/14 (64.29%) individuals with past DHF had heterotypic infections. Neut50 titres were found to be significantly higher for DENV1 than DENV2 (*p* = 0.0006) and DENV4 (*p* = 0.0127), in those with past DHF, whereas there was no significant difference seen in titres for different DENV serotypes in those with past DF. Overall NS1‐Ab to all serotypes and NS1‐specific IgG1 responses for DENV1, 2 and 4 serotypes were significantly higher in those with past DHF than individuals with past DF. Those with past DHF also had higher IgG1 than IgG3 for DENV1 and DENV3, whereas no differences were seen in those with past DF. Over 50% of those with past DF or DHF had NS1‐specific Bmem responses to >2 DENV serotypes. There was no difference in the frequency of Bmem responses to any of the DENV serotypes between individuals with past DF and DHF. Although the frequency of Bmem responses to DENV1 correlated with DENV1‐specific NS1‐Abs levels (Spearman *r* = 0.35, *p* = 0.02), there was no correlation with other DENV serotypes. We found that those with past DF had broadly cross‐reactive Nabs, while those with past DHF had higher NS1‐Ab responses possibly with a different functionality profile than those with past DF. Therefore, it would be important to further evaluate the functionality of NS1‐specific antibody and Bmem responses to find out the type of antibody repertoire that is associated with protection against severe disease.

## Graphical abstract

Those with past dengue fever (DF) had broadly cross‐reactive neutralizing antibodies, while those with past DHF had higher NS1‐specific antibody responses possibly with a different functionality profile than those with past DF. Therefore, it would be important to further evaluate the functionality of NS1‐specific antibody and memory B‐cell responses to find out the type of antibody repertoire that is associated with protection against severe disease.

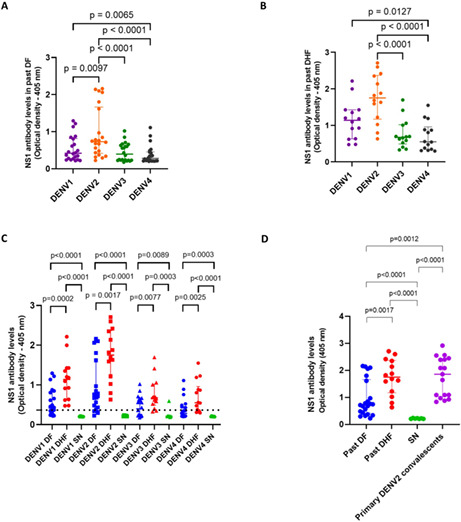

## INTRODUCTION

Infections due to dengue virus (DENV) are the leading mosquito borne viral infections globally, and due to the global burden of infection, the World Health Organization named it as one of the top 10 threats to global health in year 2019 [1]. The age‐standardized incidence rates, mortality rates and disability adjusted life years (DALYs) have risen markedly from 1990 to 2017 [2]. For instance, there has been a 107.6% global increase in DALYs from 1990 to 2017 [2]. As there is no specific treatment for dengue, currently, all patients who present with dengue fever (DF) are carefully monitored for early detection of complications for timely interventions.

Although the majority of individuals infected with the DENV develop asymptomatic or mild illness, a proportion of individuals develop dengue haemorrhagic fever (DHF), which is characterized by plasma leakage [3]. Endothelial dysfunction, which leads to plasma leakage can be due to many factors such as DENV NS1 antigen, an impaired and dysfunctional antiviral response, antibodies (Abs) specific to the previous DENV serotype enhancing infection, mast cell degranulation and an aberrant response by monocytes [4, 5, 6]. Of these many factors that cause endothelial dysfunction, the NS1 is emerging as one of the most important factors that lead to endothelial dysfunction. NS1 is secreted from infected cells and circulates as a barrel shaped hexamer, with a lipid core in the middle [7]. NS1 has shown to directly disrupt the endothelial glycocalyx layer, cause disassembly of the intercellular gap junctions, activate immune cells to produce proinflammatory cytokines, activate complement, activate monocytes to produce prostaglandins and inflammatory phospholipase A2 enzymes, and also induce the production of immunosuppressive cytokines [4, 8, 9, 10, 11]. It has been shown that high levels of NS1 during early illness is associated with progression to severe disease in some studies, although the NS1 levels and positivity rates during early illness have shown to change with the infecting DENV serotype [12, 13, 14].

While most individuals infected with the DENV do not develop severe dengue, secondary dengue infections, where an individual is infected with a subsequent different serotype than the initial infection, have shown to be an important risk factor for severe illness [15, 16]. Severe disease during secondary dengue is thought in many cases to be associated with antibody‐dependent enhancement (ADE), where poorly neutralizing Abs specific to the previous DENV serotype facilitate infection into FcγR bearing immune cells and thereby facilitate infection [17, 18]. Although NS1 has shown to contribute to severe disease by many mechanisms, in vitro and in mouse models, there are limited data in humans. For instance, the NS1 antigen levels and viral loads are lower in patients with secondary dengue than in those with primary dengue, despite secondary dengue being a risk factor for severe disease [19, 20] [21]. Therefore, there are inconsistencies in data regarding the relationship between NS1 antigen levels clinical disease severity. Therefore, in addition to the possible direct pathogenic effects of NS1 antigen, NS1 antigen–antibody complexes may also play a role in disease pathogenesis. NS1‐specific Abs have shown to cause disease pathogenesis by cross reacting with many different host‐proteins such as endothelial cells, fibrinogen and platelets [22]. NS1‐Abs have shown to bind to the endothelium, resulting in expression of ICAM‐1 and release of many proinflammatory cytokines and have shown to contribute to liver damage in murine models [22]. NS1‐Abs specific for the amino acid residues 311–330 of NS1 antigen have shown to bind to protein disulphide isomerase on platelets, thereby contributing to thrombocytopenia [23]. In contrast, NS1‐Ab have also shown to be protective in murine models and it has shown that functionality of the NS‐Abs are important in protection [24].

We previously showed that the NS1‐specific antibody levels significantly increased in those who progressed to develop DHF compared to those with DF in secondary dengue infections [25]. We also showed that those with acute DF had a higher frequency of antibody responses to different regions of NS1 than in those with DHF [25]. It was recently shown that individuals who subsequently developed inapparent dengue due to DENV3 had higher baseline levels of Abs to NS1 and envelope protein with more functional antibody responses [24]. Therefore, the antibody levels to NS1, the levels of neutralizing Abs, cross reactivity of Abs and memory B‐cell responses, all could contribute to disease severity or protection. In this study, we compared the neutralizing antibody levels to different DENV serotypes, NS1‐Ab levels, the IgG antibody subclass profiles and NS1‐specific memory B‐cell responses in healthy individuals, with varying severity of past dengue, to further understand the differences in the NS1 antibody repertoire that can lead to severe disease in dengue.

## MATERIALS AND METHODS

### Study participant recruitment and sample collection

Healthy adult individuals (*n* = 43) were recruited for the study following informed consent. Individuals who had been previously hospitalized and had been diagnosed as having DHF due to the presence of pleural effusions or ascites with platelet counts <100 000/cells^3^ based on WHO 2011 guidelines [26] were considered to have past DHF (*n* = 14). Those who were hospitalized but did not have DHF, were considered to have DF (*n* = 11) and those who were seropositive for the DENV but never hospitalized for a febrile illness were considered to have inapparent dengue/undifferentiated DF (*n* = 11). Individuals who had never been hospitalized for a febrile illness and were seronegative for the DENV by ELISA, were considered as DENV naïve individuals (*n* = 7). In those with past DHF, the time since illness was a median of 4.5 years (IQR 2.375–11.75), in those with DF, the time since illness was a median of 6 years (IQR 2–9), and is shown in Tables S1 and S2. There was no significant difference between the duration of past dengue infection in those with DF or DHF (*p* = 0.52). The year of infection in those who had an inapparent dengue infection is not known. In those who had a symptomatic infection, their infecting DENV serotype is not known as it had not been assessed at that time.

Ethical approval was obtained from the Ethics Review Committee of the Faculty of Medical Sciences, University of Sri Jayewardenepura, Sri Lanka.

#### ELISA to determine serostatus to the DENV

Panbio Dengue Indirect IgG ELISA (Panbio, Brisbane, Australia) was carried out to identify the seropositivity to the DENV. The assay was carried out according to the manufacturer's instructions and accordingly, those who had PanBio units (indirect measure of DENV IgG) of >11 were considered positive, 2–11 was considered equivocal and <2 was considered negative as previously proposed in a research carried out by Lopez et al. [27], in order to eliminate false‐negatives. Based on these interpretations, 11/18 were classified as seropositive, and the remaining 7 were considered seronegative individuals.

### Foci reduction neutralization test (FRNT) to determine neutralizing antibody titres

Neutralizing antibody titres to DENV1 WestPac74, DENV2 S16803, DENV3 CH53489 and DENV4 TVP‐376 (donated by Prof. Aravinda de Silva) were assessed using FRNTs as previously described [28]. The viruses were propagated in C6/36 cell lines, after which foci forming assays (FFAs) were carried out on Vero‐81 cells to determine the virus concentration in FFU/mL (foci forming units/mL). These viruses were used to assess the neutralizing Abs using FRNTs. Vero‐81 cells were seeded in 96‐well tissue culture treated plates and incubated overnight. The patient sera were diluted at a four‐point dilution series of 1:10, 1:40, 1:160 and 1:640 in DMEM (Gibco, Life Technologies, USA) supplemented with 2% FBS and was incubated for an hour with the calculated amount of each DENV separately. The diluted serum and virus mix was then added to the Vero‐81 cell monolayer after which the plates were incubated for an hour at 37°C with 5% CO_2_. Then, overlay media (Carboxymethyl cellulose) was added to the monolayer and the plates incubated at 37°C with 5% CO_2_ for 2–3 days. Wells were then fixed with 4% paraformaldehyde (Alfa Aesar, UK) and blocked with blocking buffer (1X perm buffer (Biolegend, USA) with 3% normal goat serum (Sigma, USA)). Staining was carried out using a mixture of 4G2 and 2H2 biotinylated Abs (donated by Prof. Aravinda de Silva). HRP conjugated goat anti‐mouse IgG (KPL, SeraCare Life Sciences, USA) was used as the secondary antibody, followed by TrueBlue Peroxidase substrate (KPL, SeraCare Life Sciences, USA). The assays were performed in duplicate.

The RStudio software was used to count the spots and GraphPad Prism version 9 was used to analyse data and to calculate Neut_50_ by plotting neutralization percentages against the log (1/dilution) values. Those with Neut_50_ titres <10 for all 4 serotypes was classified as naïve individuals, those who had Neut_50_ titres ≥10 for only one serotype, or ≥10 for different serotypes but 5 times higher for one serotype only were classified as having a monotypic antibody profile, and those with Neut_50_ titres ≥10 for different serotypes (without higher titres for a single serotype) were considered as having a heterotypic antibody profile.

### Determining levels of NS1‐specific antibody levels for different DENV serotypes

In order to determine the NS1 antibody levels specific to each serotype of DENV in the sera of these individuals, an in‐house indirect ELISA was performed as described previously [25]. Briefly, 96‐well plates were coated with DENV1, DENV2, DENV3 and DENV4 NS1 recombinant proteins expressed in mammalian HEK293 cells (Native Antigen, USA), separately. The plates were blocked with blocking buffer (PBS containing 0.05% Tween 20 and 1% Bovine serum albumin [BSA]). The serum samples that were diluted at 1:5000 were added to the plates and the ELISA was developed using goat anti‐human IgG biotinylated antibody (Mabtech, Sweden), Streptavidin Alkaline Phosphatase (Abcam, UK) as the enzyme and Para‐nitro‐phenyl‐phosphatase (PNPP) (Thermofisher Scientific, USA) as the substrate. Absorbance was measured at 405 nm using the MPSCREEN MR‐96A ELISA reader. The positive cut‐off threshold was set at mean ± SD of the optical density (OD) value of seronegative individuals. Although we sought to find out the NS1‐antibody levels in individuals with primary dengue due to different serotypes, we could only do this for DENV2 as we had not detected any person with a primary dengue infection due to other serotypes in the past 5 years. Therefore, to find out a positive cut‐off point only for DENV2, samples collected on day 14–21 (*n* = 17) from individuals infected with primary DENV2 infections were used to compare with DENV2 NS1‐specific responses of those with past infections.

### Detection of NS1‐specific IgG1 and IgG3 antibody levels in sera

Levels of NS1‐specific IgG1 and IgG3 subclass to NS1 of all 4 DENV serotypes was assessed individually in serum samples using an in‐house ELISA. The 96‐well ELISA plates were coated at 5 μg/mL with DENV1, DENV2, DENV3 and DENV4 NS1 proteins (Native Antigen, USA), separately. The wells were then blocked and serum was diluted at 1:250, which was added to the plate. IgG1 and IgG3 levels were detected using biotinylated goat anti‐human IgG1 (Mabtech, Sweden, Cat: 3851‐6‐250) and biotinylated goat anti‐human IgG3 (Mabtech, Sweden, Cat: 3853‐6‐250), respectively. Streptavidin‐horse radish peroxidase (Mabtech, Sweden, Cat: 3310‐9) and TMB (Mabtech, Sweden, Cat: 3652‐F10) substrate was used to develop the reaction. The absorbance values were obtained at 450 nm using the MPSCREEN MR‐96A ELISA reader.

### Memory B‐cell ELISpot to determine Bmem responses to DENV NS1 protein

Memory B‐cell Responses (Bmem) specific for DENV1, DENV2, DENV3 and DENV4 NS1 proteins were assessed as previously described by us for assessing Bmem responses to different COVID‐19 vaccines [29, 30]. Briefly, freshly isolated PBMCs were stimulated in a 24 well plate using IL‐2 and R848 (a TLR 7/8 agonist) in RPMI media (Gibco, Life Technologies, USA) supplemented with 10% Fetal Bovine Serum, 1% Penicillin Streptomycin and 1% Glutamine (Gibco, Life Technologies, USA) (R10 media) and incubated at 37°C with 5% CO_2_ for 3 days. Unstimulated PBMCs were also incubated in R10 media, as a negative control. After an incubation period of 3 days, the cells were rested overnight. The assay was optimized by adding 100,000 cells/well and the optimal coating concentration was determined to be 1 μg/mL. 50,000 cells/well were added to the positive control wells. A Human IgG ELISpot BASIC kit (Mabtech 3850‐2A) was used according to the manufacturer's guidelines to quantify IgG‐secreting cells specific to DENV NS1 full length recombinant proteins (Native Antigen, UK) for all four serotypes, which was used to coat the wells at 1 μg/mL in PBS. Anti‐human IgG monoclonal capture Abs were used to coat the positive control wells and culture media was used for the negative control. The experiments were carried out in duplicate and the spots were counted using the automated AID iSpot Reader System (GmbH Germany). Mean ± 2 SD of the background responses was defined as a positive response.

### Statistical analysis

Statistical analysis was carried out using the GraphPad Prism version 9. As the data were not normally distributed, non‐parametric tests were carried out. All tests were two‐tailed. The Mann–Whitney *U* test was performed to compare differences in antibody levels, and the frequency of Bmem between those with past DHF, DF and DENV seronegative individuals. Differences in FRNT responses, NS1‐antibody levels and Bmem responses between serotypes were compared using the Friedman test along with a Dunns multiple comparison *t* test to compare between serotypes. The Wilcoxon's matched pairs signed rank test was used to compare paired data when responses to IgG1 and IgG3 were compared between the same individuals. Spearman's rank two‐tailed correlation coefficient was used to evaluate the correlation between variables including Bmem responses, antibody levels and Nabs levels (Neut_50_ titres).

## RESULTS

### Neutralizing antibody (Nab) levels in individuals with varying severity of past dengue infections

Out of the 43 healthy individuals that were enrolled in the study, 28/43 (65.1%) were females and 15/43 (34.88%) were males. Their median age was 33 years (IQR 29–40 years). The Nab levels were assessed in healthy individuals with past DHF (*n* = 14), those with past DF (*n* = 11), those with inapparent dengue/undifferentiated DF (*n* = 11) and in DENV seronegative individuals (*n* = 7). In those with past DHF, the time since illness was a mean of 7.18 years, and in those with DF the time since illness was a mean of 7.32 years (Tables S1 and S2). No significant difference was observed in the time since illness of those with DF when compared to those with DHF (*p* = 0.847). The time since infection in those with undifferentiated DF was not known, as they did not know when they were infected. Those who were hospitalized due to DF and had undifferentiated DF, were classified together as having past DF (*n* = 22) during the analysis. The Nab levels were expressed as Neut50 titres, indicating the Nabs levels at which 50% neutralization of the virus was achieved.

Of those with past DF, 15/22 (68.18%) had Nabs to several DENV expressing heterotypic antibody profiles, 5/22 (22.72%) only had responses to one DENV serotype indicating monotypic antibody profiles and 2/22 (9.09%) did not have detectable Nabs despite being seropositive by the ELISA. In those with past DHF, heterotypic antibody profiles were seen in 9/14 (64.29%) individuals and monotypic antibody profiles in 5/14 (35.71%). In individuals with past DF, 8/22 (36.36%) had Nabs against 4 serotypes (Table 1), with 15/22 (68.18%) having Nabs against DENV1, 16/22 (72.73%) against DENV2, 15/22 (68.18%) against DENV3 and 12/22 (54.55%) against DENV4. In those who had past DHF infections, Nabs against all 4 serotypes were present in 7/14 (50%) individuals, with all 14/14 (100%) having Nabs against DENV1, 7/14 (50%) having Nabs against DENV2, and 9/14 (64.29%) against DENV3 and DENV4 (Table 1). The seven individuals, who were determined as seronegative for DENV using the IgG Indirect ELISA, were also naïve for neutralizing Abs against all four DENV serotypes.

**TABLE 1:** Neutralizing antibody (Neut50) titres in individuals with past dengue fever (DF) and past dengue haemorrhagic fever (DHF) against all 4 DENV serotypes.

	Neutralizing antibody (Neut50) titres			
	DENV1	DENV2	DENV3	DENV4
Individuals with past DF				
DF1	187.76	388.06	306.20	187.76
DF2	399.39	329.69	183.02	130.41
DF3	15.16	603.39	136.36	27.91
DF4	0	0	0	0
DF5	48.70	148.18	100	0
DF6	181.26	640	129.99	226.78
DF7	13.21	640	47.20	42.03
DF8	51.42	28.55	21.13	175.55
DF9	113.99	26.19	32.23	71.93
DF10	70.79	158.49	209.31	83.33
DF11	0	0	0	0
DF12	0	0	0	32.33
DF13	0	39.30	106.95	0
DF14	42.58	0	33.82	56.23
DF15	34.36	139.44	0	26.44
DF16	55.51	60.72	180.67	26.95
DF17	14.96	183.61	0	0
DF18	296.48	0	33.17	16.63
DF19	105.92	30.24	37.71	12.35
DF20	384.33	170.57	21.34	0
DF21	640	10.76	10.26	45.53
DF22	416.29	125.49	0	13.04
Individuals with past DHF				
DHF1	640	21.47	15.85	14.31
DHF2	261.88	38.31	27.21	39.68
DHF3	413.71	0	0	47.77
DHF4	558.73	0	141.25	122.72
DHF5	209.31	26.52	132.92	28.18
DHF6	198.24	0	119.23	93.20
DHF7	542.88	1.25	50.12	58.06
DHF8	486.97	14.44	46.57	93.20
DHF9	264.36	43.82	111.84	58.06
DHF10	640	185.40	199.53	90.55
DHF11	576.90	329.69	118.47	19.57
DHF12	16.63	229.67	92.32	132.07
DHF13	59.57	44.81	212.03	84.43
DHF14	640	252.81	313.18	84.41

**FIGURE 1:**
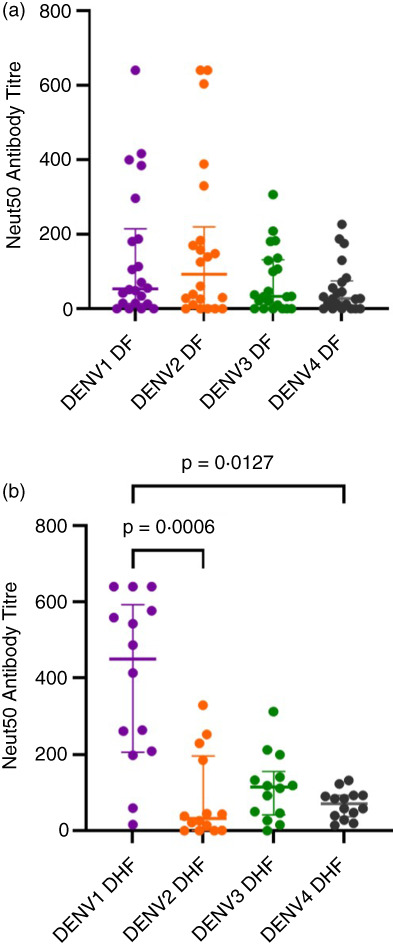
Neutralizing antibody levels (Neut50 titres) to DENV1, DENV2, DENV3 and DENV4 in those with varying severity of past dengue infections. Neutralizing antibody (Nab) levels were measured by FRNTs in those who had past DF (*n* = 22) and past DHF (*n* = 11). The differences in the Nab (Neut50 titres) between different DENV serotypes were analysed in those with past DF (a) and past DHF (b) using the Friedman test. All tests were two sided. The lines represent the median and the interquartile range.

The Nabs levels (Neut50 titres) were found to be significantly higher for DENV1 than DENV2 (*p* = 0.0006) and DENV4 (*p* = 0.0127) in those with past DHF, whereas there was no significant difference between the Neut50 titres for different DENV serotypes in those with past DF (Figure 1a,b). The Nabs for DENV1 (*p* = 0.0006) and DENV4 (*p* = 0.0436) were significantly higher in those with past DHF compared to those with past DF. Although not significant (*p* = 0.4241), the Neut50 titres were higher for DENV2 (median 93.1, IQR 8.07–220.1) in those with past DF compared to past DHF (median 33.42, IQR 0.94–196.5).

### Comparison of serotype‐specific NS1 antibody responses in those with varying severity of past dengue

**FIGURE 2:**
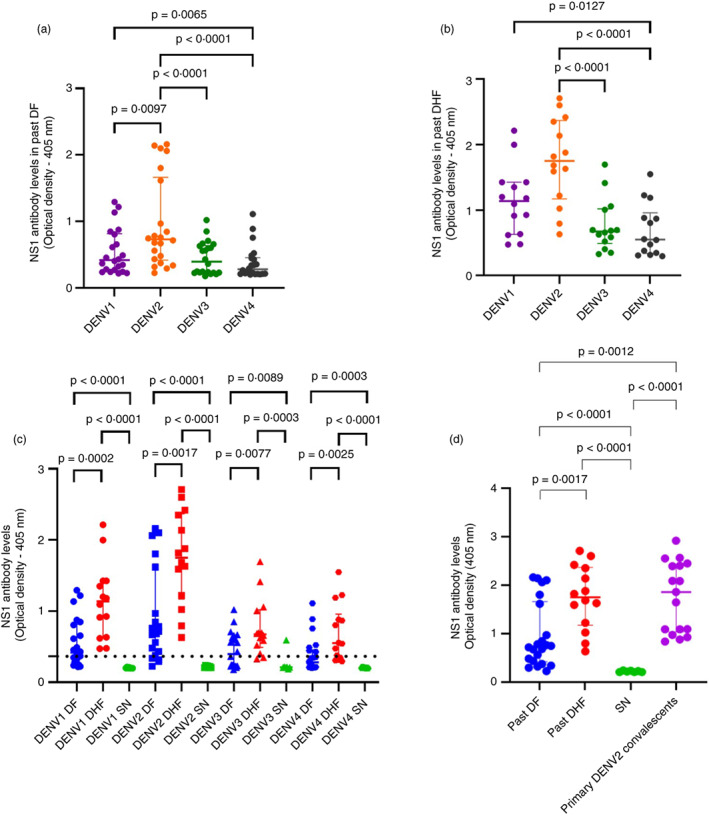
NS1‐specific antibody responses to the four DENV serotypes in those with varying severity of past dengue infections. NS1‐specific antibodies (Abs) to the DENV serotypes were assessed by an in‐house ELISA in those with past DF (*n* = 22), those with past DHF (*n* = 14) and seronegative individuals (*n* = 7). Comparison of NS1‐Ab responses between the serotypes in those with past DF (a) and past DHF (b) were analysed using the Friedman test. The differences between NS1‐specific antibody responses in individuals with past DF, past DHF and seronegative (SN) individuals were analysed using the Mann–Whitney *U* test (c). Comparison of DENV2 NS1‐Ab responses in those with past DF, those with past DHF and seronegative (SN) individuals with the DENV2 NS1‐specific Ab responses of convalescent samples of individuals with primary DENV2 infections (*n* = 17) were analysed using the Mann–Whitney *U* test (d). All tests were two‐tailed. The horizontal dotted line represents the positive cut‐off (mean ± 2 SD of the background responses). The lines represent the median and the interquartile range.

We previously showed that NS1‐Ab responses were significantly higher in those with past severe dengue, but we had only investigated NS1‐Ab responses to one DENV serotype [25]. Therefore, to determine the relationship between NS1‐Ab responses to different serotypes and the extent of cross reactivity, we assessed the NS1‐Ab levels to all four serotypes in individuals with past DHF or DF and in seronegative individuals. Both individuals with past DF and past DHF had the highest antibody levels for NS1 of DENV2. In those with past DF, Ab levels against DENV2 NS1 were significantly higher than DENV1 (*p* = 0.0097), DENV3 (*p* < 0.0001) and DENV4 (*p* < 0.0001) (Figure 2a). A similar pattern was seen in those with past DHF, where NS1‐Ab levels against DENV2 were significantly higher than DENV3 (*p* < 0.0001) and DENV4 (*p* < 0.0001), and NS1‐Ab levels against DENV1 was significantly higher than DENV4 (*p* = 0.0127) (Figure 2b).

NS‐1Ab responses in those with past DHF were significantly higher than individuals with past DF for all four DENV serotypes (DENV1, *p* = 0.0002; DENV2, *p* = 0.0017; DENV3, *p* = 0.008 and DENV4, *p* = 0.003) (Figure 2c). DENV3‐specific NS1‐Ab responses were seen in one seronegative individual (Figure 2c), although this individual did not have any Nabs to any of the DENV serotypes. The DENV2‐specific NS1 Ab responses of individuals with past DF and past DHF were compared with individuals who had primary DENV2 infections, showing that individuals with past DF had significantly lower NS1 Ab responses than those who had primary DENV2 infection (*p* = 0.0012) (Figure 2d). However, there was no significant difference between the responses of those with DHF and those with the known DENV2 infection (*p* = 0.9532) (Figure 2d).

The NS1‐Ab responses positively and significantly correlated with the neutralizing antibody levels (Neut50 titres in all individuals [*n* = 43]) to DENV1 (Spearman *r* = 0.69, *p* < 0.0001), DENV2 (Spearman *r* = 0.53, *p* = 0.0003), DENV3 (Spearman *r* = 0.62, *p* < 0.0001) and DENV4 (Spearman *r* = 0.68, *p* < 0.0001) (Figure S1).

### Comparison of NS1 IgG1 and IgG3 subclass‐specific Abs in those with varying severity of past dengue

We previously reported that NS1‐Abs of patients with acute DF predominantly recognized different regions of the protein than those with acute DHF [25]. However, apart from the specificity of NS1‐Abs and quantitative differences of NS1‐Abs in individuals with varying severity of illness, the functionality of NS1‐Abs could differ, thereby contributing to disease pathogenesis. As the Fc portion of an antibody is shown to be critical in immune complex formation, inducing antibody‐dependent cell mediated cytotoxicity (ADCC) by NK cells, and has the ability to activate phagocytes and complement [31, 32], we assessed the levels of NS1‐specific IgG1 and IgG3 responses for all four DENV serotypes in our cohort.

**FIGURE 3:**
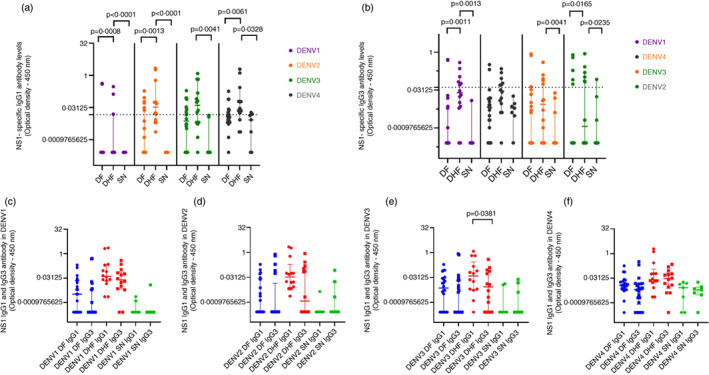
NS1‐specific IgG1 and IgG3 responses to the four DENV serotypes in those with varying severity of past dengue infections. NS1‐specific IgG1 and IgG3 antibody levels were measured for the four DENV serotypes in individuals with past DF (*n* = 22), those with past DHF (*n* = 14) and seronegative (SN) individuals (*n* = 7) using an in‐house ELISA. Comparison in IgG1 responses (a) and IgG3 responses (b) between those with past DF, DHF and seronegative individuals were analysed using the Mann–Whitney *U* test. The horizontal dotted line represents the positive cut‐off (mean ± 2 SD of the background responses). Comparison between IgG1 and IgG3 responses in those with past DF, past DHF and seronegative individuals to DENV1 (c), DENV2 (d), DENV3 (e) and DENV4 (f) were analysed using the Wilcoxon signed rank test. All tests were two‐tailed. The lines indicate median and the interquartile range.

Those with past DHF had significantly higher NS1‐specific IgG1 responses to DENV1 (*p* = 0.0008), DENV2 (*p* = 0.0013) and DENV4 (*p* = 0.0328) than those with past DF (Figure 3a). Those with past DHF also had higher NS1‐IgG3 antibody responses to DENV1 (*p* = 0.0011) and DENV4 (*p* = 0.0165) compared to those with past DF and seronegative individuals (Figure 3b). Individuals with past DHF had significantly higher NS1‐IgG1 Ab responses than NS1 IgG3 for DENV1 and DENV3 although this difference was only significant for DENV3 (*p* = 0.038) but not for any other DENV serotypes (Figure 3c–f). There was no difference between the NS1‐IgG3 Ab responses and NS1‐IgG1 Ab responses in those with past DF.

### Memory B‐cell (Bmem) responses to NS1 in those with varying severity of past dengue

**FIGURE 4:**
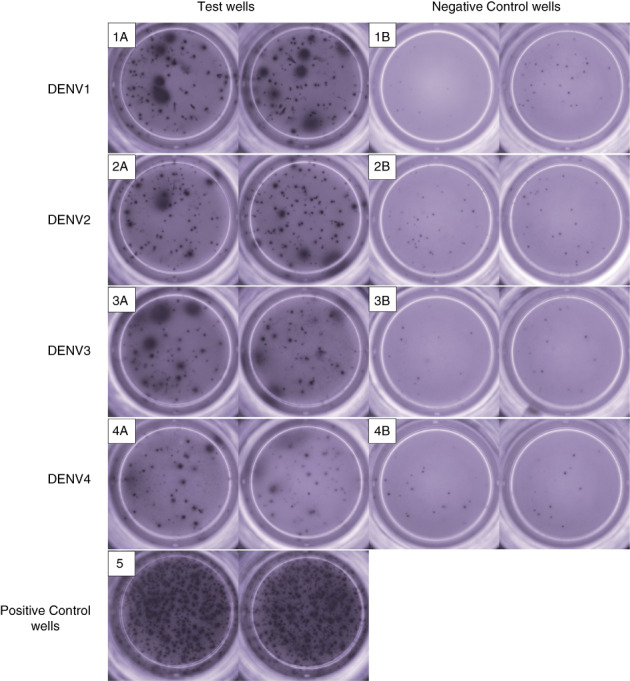
NS1‐specific memory B‐cell responses (Bmem) to all four DENV serotypes in an individual with past DHF. The Bmem responses were measured by B‐cell ELISpot assays to DENV NS1 for DENV1 (1A), DENV2 (2A), DENV3 (3A) and DENV4 (4A), and responses to culture media, which was used as negative controls (1B, 2B, 3B and 4B). The positive control wells show the total IgG‐secreting cells (5) for the same individual. Each test was been carried out in duplicate.

As Bmem responses to DENV NS1 have not been assessed before, we sought to investigate the frequency of Bmems in our cohort for different DENV serotypes and their association with NS1‐Ab levels and Nabs. Bmem responses to each serotype and their respective negative controls are represented in Figure 4. Of the 43 individuals who were assessed for DENV NS1‐specific Bmem responses, responses for ≥3 serotypes were seen in 9/22 (40.91%) individuals with past DF and 7/14 (50%) individuals with past DHF (Table 2). Of the DENV seronegative individuals, 2/7 (28.57%) had Bmem responses to 2 serotypes (one for DENV1 and DENV4, and the other person to DENV1 and DENV2), while the remaining 5/7 (71.43%) did not respond to any of the 4 serotypes. These seronegative individuals did not have any responses to DENV by the FRNT assay and did not give a positive response for the presence of NS1‐Abs to any of the DENV serotypes.

**TABLE 2:** Frequency of NS1‐specific memory B‐cell responses (Bmem) in individuals with past dengue fever (DF), past dengue haemorrhagic fever (DHF) and in seronegative individuals to NS1 antigen of all for DENV serotypes. The values are expressed as antibody secreting cells (ASCs) per million cells.

	Bmem responses (ASCs/1 million cells)			
	DENV1	DENV2	DENV3	DENV4
Individuals with past DF				
DF1	20	5	5	0
DF2	30	25	50	20
DF3	60	85	65	60
DF4	0	45	15	80
DF5	10	10	0	10
DF6	10	15	20	25
DF7	30	55	35	25
DF8	60	65	30	20
DF9	545	870	0	160
DF10	50	75	55	70
DF11	65	150	45	0
DF12	50	50	25	20
DF13	30	25	20	50
DF14	10	0	10	5
DF15	125	50	50	0
DF16	10	10	0	5
DF17	95	155	120	50
DF18	630	795	195	185
DF19	375	585	370	165
DF20	270	155	70	35
DF21	190	135	25	55
DF22	25	50	0	0
Individuals with past DF				
DHF1	930	415	430	355
DHF2	30	40	15	20
DHF3	520	450	210	210
DHF4	175	105	125	85
DHF5	215	145	70	60
DHF6	20	15	15	0
DHF7	120	35	20	10
DHF8	10	0	15	0
DHF9	50	30	20	65
DHF10	685	530	85	245
DHF11	60	115	105	5
DHF12	5	5	25	5
DHF13	45	170	10	15
DHF14	325	310	50	185
Seronegative individuals				
SN1	0	0	5	5
SN2	90	65	35	10
SN3	105	40	50	50
SN4	5	5	10	5
SN5	15	10	0	20
SN6	0	5	5	0
SN7	25	15	5	10

**FIGURE 5:**
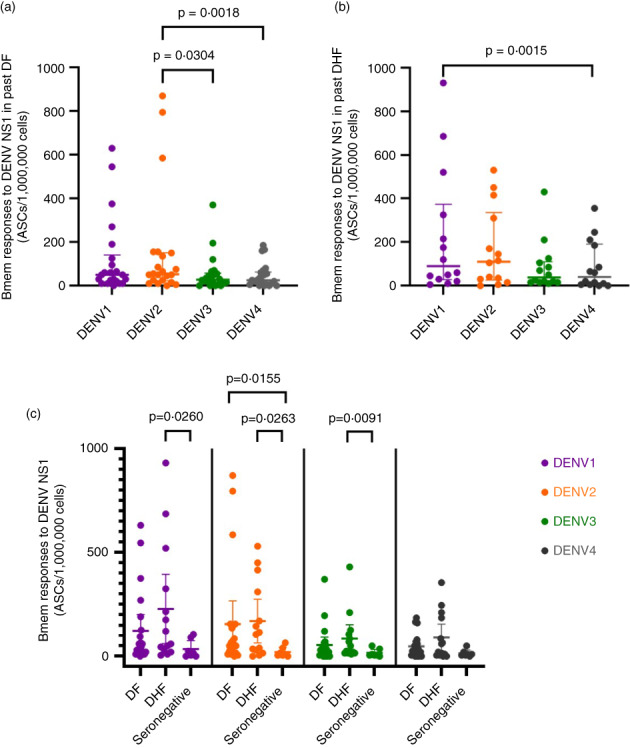
The frequency of NS1‐specific memory B‐cell (Bmem) responses to the four DENV serotypes in individuals with varying severity of past dengue infections. The Bmem responses were measured by B‐cell ELISpot assays in those who had past DF (*n* = 22), past DHF (*n* = 11) and seronegative individuals for the four DENV serotypes (SN) (*n* = 7). The differences in the frequency of Bmem responses for four DENV serotypes were analysed in those with past DF (a) and past DHF (b) using the Friedman test. Comparisons in NS1‐specific antibody responses between individuals with past DF, past DHF and seronegative (SN) individuals were analysed using the Mann–Whitney *U* test (c). All tests were two‐tailed. The lines indicate median and the interquartile range.

In those with past DF, the frequency of Bmem responses for DENV2 were significantly higher than DENV3 (*p* = 0.0304) and DENV4 (*p* = 0.018) (Figure 5a), whereas in those with past DHF, Bmem responses were significantly higher against DENV1 than DENV4 (*p* = 0.0015) (Figure 5b). There was no difference in the frequency of Bmem responses to any of the DENV serotypes between individuals with past DF and DHF (Figure 5c). A positive correlation was seen for DENV1 responses (Spearman *r* = 0.50, *p* = 0.0006) with the frequency of DENV‐specific Bmem responses, but no correlation was seen between responses to DENV2 (B), DENV3 (C) and DENV4 (D) (Figure S2).

## DISCUSSION

In this study we assessed the exposure to different DENV serotypes using the FRNT assay and compared the Nab levels, NS1‐Ab levels, NS1‐Ab levels for IgG1 and IgG3 and Bmem responses to all four DENV serotypes in individuals with varying severity of past infection. We found that there was no difference between individuals having heterotypic antibody profiles with past DF (64.29%) and DHF (68.18%), suggesting that an equal proportion of those with secondary dengue infection, experienced milder forms of dengue and DHF. Since both, those with past DF and DHF were infected many years ago, these levels do not reflect the NS1‐Ab levels or Bmem responses to the DENV or NS1 at the time of acute infection or recovery. In addition, these individuals could have had an asymptomatic or mild dengue infection between the symptomatic episode of DF or DHF, which could in fact affect the Nabs levels, NS1‐antibody levels and Bmem responses. Nevertheless, many years following infection, significant differences in the NS1‐Ab levels with differences in IgG subtypes were seen in those with varying severity of past infection.

In a large longitudinal study in a community cohort, it was shown that both primary and secondary dengue infections were equally likely inapparent and that the Nabs were lower in those who developed symptomatic dengue [33]. In our cohort, those with past DF had equal levels of Nabs for all four DENV serotypes, while those with past DHF had significantly higher levels to DENV1 and DENV4. Individuals with past DHF had significantly higher Nabs levels to DENV1 but higher levels of NS1 antibody levels to DENV2. Higher levels of Nabs to DENV1 could be due to primary infection with DENV1 serotype followed by a secondary DENV2 infection. Sri Lanka had DENV1 as the predominant serotype from 2009 to mid‐2016 and DENV2 as the predominant serotype from mid‐2016 onwards [13, 34]. Therefore, it is likely that the primary infection due to DENV1 could have led to higher Nab for DENV1. Although not significant both Nab and NS1‐Abs were lowest to DENV4. Interestingly, Sri Lanka has not reported an outbreak due to DENV4, and we have not identified this serotype in hospitalized patients during our routine surveillance activities [35]. As those with past DF had broadly cross‐reacting Nabs, the breadth of the Nabs could influence clinical disease severity, when infected with different serotypes of the DENV.

Those with past DHF had higher NS1‐Abs levels for all four DENV serotypes than those with past DF. The higher NS1‐Ab levels could be due to higher NS1 antigen levels during acute illness, which has been reported in those who progress to develop DHF, although the NS1‐Ab levels tested do not reflect the values at the time of acute infection [12]. Interestingly, in contrast to the observations with Nab levels, the NS1‐Ab levels were highest for DENV2 in individuals with both past DF and DHF. However, in addition to the levels of Abs, the functionality of Abs are known to be important in protection or disease pathogenesis [32]. It was shown that those who had higher levels of total DENV‐specific IgG and IgG4 had inapparent dengue infection when infected with DENV3 compared to those who had lower levels [24]. Apart from neutralization of the antigen, Abs have many functions such as complement activation, facilitating killing by natural killer cells by antibody‐dependent cell mediated cytotoxicity (ADCC) and phagocytosis of antibody coated cells [32]. While both IgG1 and IgG3 bind to FcγRIIa, FcγRIIIa and FcγRIIIb, the affinity of IgG3 has shown to be higher [36]. IgG3 has shown to be critical in immune responses to many viruses and bacterial pathogens [37]. We found that those with past DHF had higher levels of NS1‐specific IgG1 than individuals with past DF to three DENV serotypes and higher NS1‐specific IgG3 to DENV1 and DENV4 than those with past DF. In addition, although those with past DHF had higher NS1‐specific IgG1 than IgG3 to DENV1 and DENV3, there was no difference between IgG1 and IgG3 levels to any of the DENV serotypes in those with past DF. These data suggest that the NS1‐specific IgG subclasses are likely to be different in those with varying severity of dengue, which would have implications for protection when re‐infected with a DENV. Therefore, it would be important to further examine the functionality of NS1‐specific antibody responses in those with acute illness and to carefully follow up longitudinal cohorts to determine the type of antibody repertoire that is associated with protection against severe disease.

Long lived memory B cells (Bmems) are important for recall responses to an antigen and the levels of circulating antibody levels, and may not necessarily reflect the presence and frequency of Bmems for a particular antigen [38]. Although Bmems for the envelope and PrM protein of the DENV has been previously studied, NS1‐specific Bmems have not been investigated before [39, 40]. We found that, as seen with FRNT assays, a large proportion of our cohort had NS1‐specific Bmem responses to two or more DENV serotypes. In those with past DF the highest frequencies of responses were seen to DENV2, whereas those with DHF had the highest responses to DENV1, as seen with NS1‐Ab responses. However, unlike seen with NS1‐Ab levels, the frequency of Bmem cells were not higher in individuals with past DHF than DF. In order to further characterize the profile of Bmem cells, it would be important to assess the IgG subclass profile and specificity of these Bmems in individuals with varying severity of dengue.

In summary, we found that those with past DF had broadly cross‐reactive Nabs, while those with DHF had low levels of Nabs to some serotypes. In contrast, those with past DHF had higher NS1‐Ab responses to three DENV serotypes compared to those with DF, and higher IgG1 responses. All individuals had NS1‐specific Bmem responses to more than one DENV serotype. It would be important to further evaluate the functionality of NS1‐specific antibody and Bmem responses to determine the type of antibody repertoire that is associated with protection against severe disease.

## AUTHOR CONTRIBUTIONS

*Study design*: Shyrar Tanussiya Ramu and Gathsaurie Neelika Malavige. *Experiments*: Shyrar Tanussiya Ramu, Madushika Dissanayake, Farha Bary, Michael Harvie, Laksiri Gomes, and Ayesha Wijesinghe. *Data analysis*: Shyrar Tanussiya Ramu and Gathsaurie Neelika Malavige. *Project administration and supervision*: Gathsaurie Neelika Malavige and Chandima Jeewandara. *Funding acquisition*: Gathsaurie Neelika Malavige, Graham S. Ogg, and Chandima Jeewandara. *Writing and reviewing the manuscript*: Shyrar Tanussiya Ramu, Gathsaurie Neelika Malavige, and Graham S. Ogg.

## CONFLICT OF INTEREST STATEMENT

The authors declare no conflicts of interest.

## Supporting information


**Figure S1.** Relationship between NS1‐specific antibody responses and neutralizing antibody levels in individuals with varying severity of past dengue infections. NS1‐specific antibody responses (*n* = 43) positively and significantly correlated with the neutralizing antibody levels (Neut50 titres) in all individuals (*n* = 43) to DENV1 (Spearman *r* = 0.69, *p* < 0.0001) (A), DENV2 (Spearman *r* = 0.53, *p* = 0.0003) (B), DENV3 (Spearman *r* = 0.62, *p* < 0.0001) (C) and DENV4 (Spearman *r* = 0.68, *p* < 0.0001) (D).

**Figure S2.** Relationship between NS1‐specific Bmem responses and neutralizing antibody levels in individuals with varying severity of past dengue infections. The frequency of memory B‐cell responses to NS1 (*n* = 43) was correlated with the frequency of neutralizing antibody titres (Neut50 titres) in all individuals (*n* = 43) to each DENV serotype. A positive correlation was seen for DENV1 responses (Spearman *r* = 0.50, *p* = 0.0006) (A), with no correlation between responses to DENV2 (B), DENV3 (C) and DENV4 (D).

**Table S1.** Year of hospitalization and number of years before sample collection in individuals with past DF.
**Table S2.** Year of hospitalization and number of years before sample collection in individuals with past DHF.

## Data Availability

The data that support the findings of this study are available from the corresponding author upon reasonable request.
