# Supplementary figures and images for: Antibody and memory B cell responses to the dengue virus NS1 antigen in individuals with varying severity of past infection
Source: Immunology. 2023 Apr 19;170(1):47–59. doi: 10.1111/imm.13651 (PMC11495261; doi:10.1111/imm.13651)

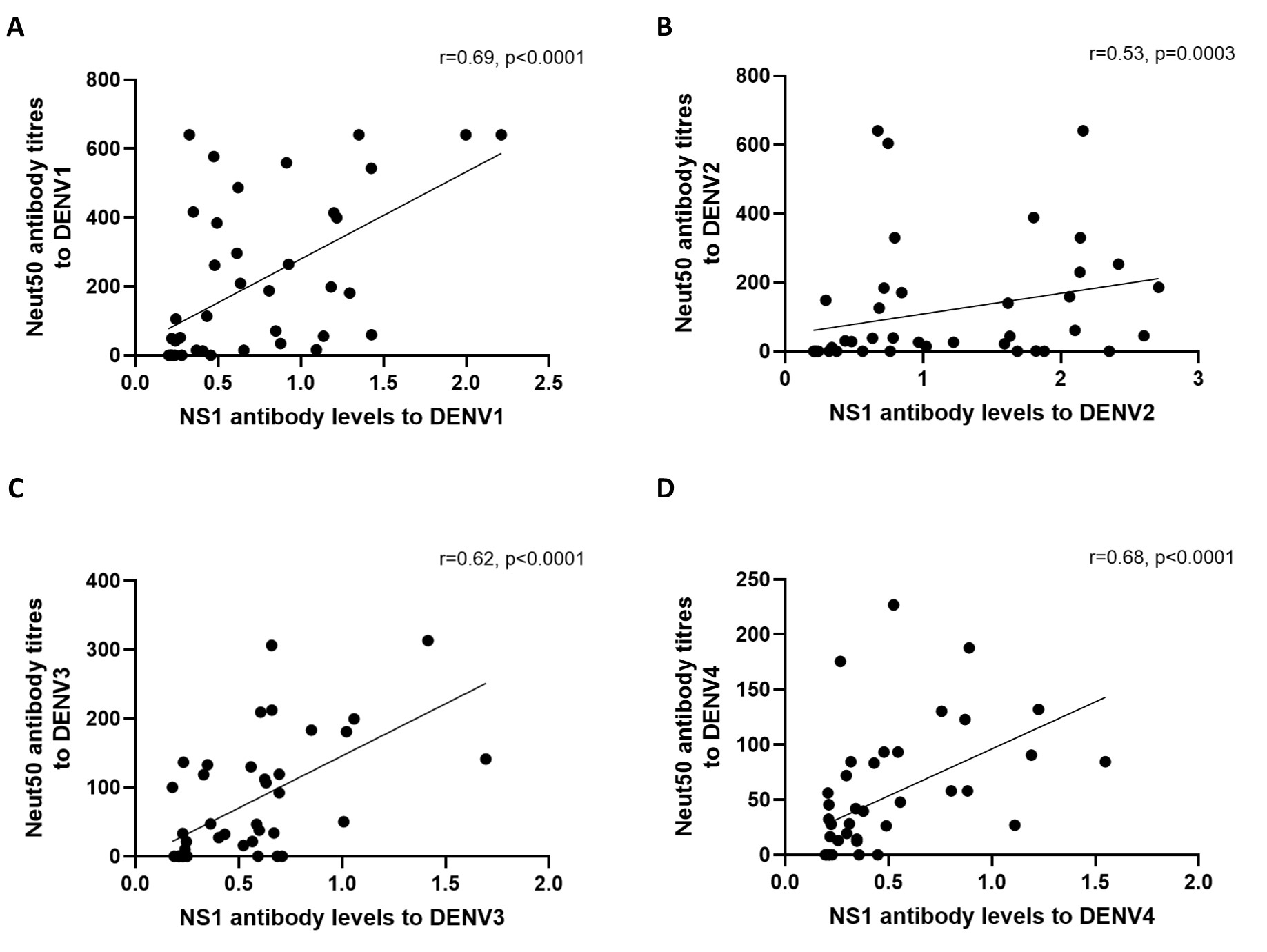

Supplement: Supplementary file 1 — Figure S1. Relationship between NS1‐specific antibody responses and neutralizing antibody levels in individuals with varying severity of past dengue infections. NS1‐specific antibody responses (n = 43) positively and significantly correlated with the neutralizing antibody levels (Neut50 titres) in all individuals (n = 43) to DENV1 (Spearman r = 0.69, p < 0.0001) (A), DENV2 (Spearman r = 0.53, p = 0.0003) (B), DENV3 (Spearman r = 0.62, p < 0.0001) (C) and DENV4 (Spearman r = 0.68, p < 0.0001) (D). [file IMM-170-47-s003.jpg]

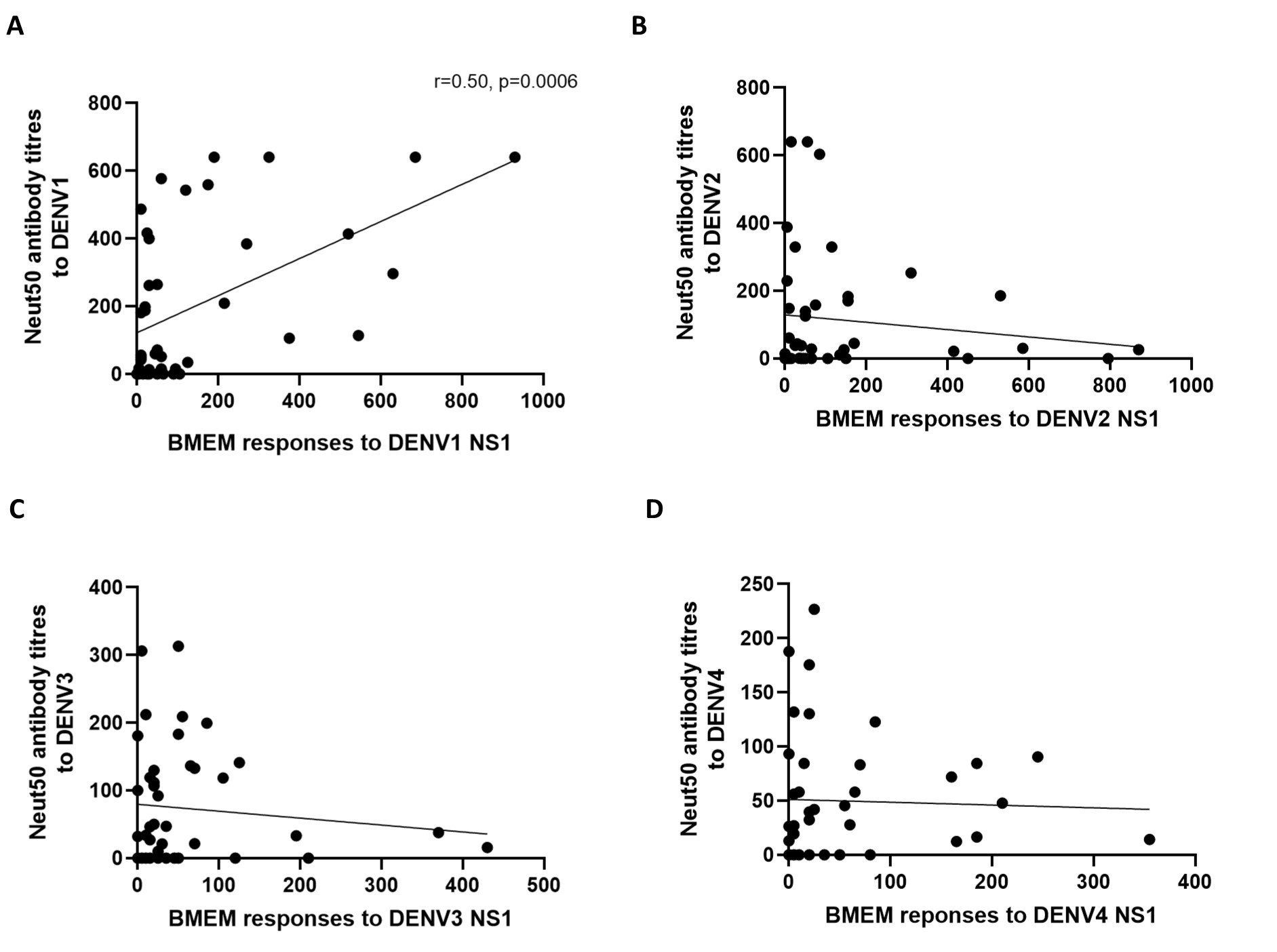

Supplement: Supplementary file 2 — Figure S2. Relationship between NS1‐specific Bmem responses and neutralizing antibody levels in individuals with varying severity of past dengue infections. The frequency of memory B‐cell responses to NS1 (n = 43) was correlated with the frequency of neutralizing antibody titres (Neut50 titres) in all individuals (n = 43) to each DENV serotype. A positive correlation was seen for DENV1 responses (Spearman r = 0.50, p = 0.0006) (A), with no correlation between responses to DENV2 (B), DENV3 (C) and DENV4 (D). [file IMM-170-47-s001.jpg]
