# Supplementary material for: Antibody and memory B cell responses to the dengue virus NS1 antigen in individuals with varying severity of past infection
Source: Immunology. 2023 Apr 19;170(1):47–59. doi: 10.1111/imm.13651 (PMC11495261; doi:10.1111/imm.13651)
Supplement: Supplementary file 3 — Table S1. Year of hospitalization and number of years before sample collection in individuals with past DF. Table S2. Year of hospitalization and number of years before sample collection in individuals with past DHF. [file IMM-170-47-s002.docx]

**Supplementary Table 1: Year of hospitalization and number of years before sample collection in individuals with past DF**

| Individuals with past DF | Year of hospitalization | Number of years before serum sample collection |
| --- | --- | --- |
| DF1 | 2000 | 21 years |
| DF2 | 2017 | 4 years |
| DF3 | 2017 | 4 years |
| DF4 | 2019 | 2 years |
| DF5 | 2004 | 17 years |
| DF6 | 2015 | 6 years |
| DF7 | 2021 | 0.5 years |
| DF8 | 2014 | 7 years |
| DF9 | 2013 | 8 years |
| DF10 | 2021 | 9 years |
| DF11 | 2019 | 2 years |

**Supplementary Table 2: Year of hospitalization and number of years before sample collection in individuals with past DHF**

| Individuals with past DHF | Year of hospitalization | Number of years before serum sample collection |
| --- | --- | --- |
| DHF1 | 2017 | 4 years |
| DHF2 | 2010 | 11 years |
| DHF3 | 2016 | 5 years |
| DHF4 | 2017 | 4 years |
| DHF5 | 2017 | 4 years |
| DHF6 | 2010 | 11 years |
| DHF7 | 2016 | 5 years |
| DHF8 | 2007 | 14 years |
| DHF9 | 2022 | 0.5 years |
| DHF10 | 2021 | 0.5 years |
| DHF11 | 2021 | 0.5 years |
| DHF12 | 2018 | 3 years |
| DHF13 | 2002 | 22 years |
| DHF14 | 2021 | 0.5 years |
